# Correction: The spatial spillover effect and its attenuation boundary of urban economy on port efficiency

**DOI:** 10.1371/journal.pone.0355262

**Published:** 2026-07-31

**Authors:** Deng Zhao, Xu Dongmei, Zhou Yutao, Daun Wei

## Notice of Republication

This article was republished on July 17^th^, 2026, to correct errors in the author’s affiliations. The author list should be Deng Zhao^1,2^, Xu Dongmei^1^, Zhou Yutao^3^, Daun Wei^3^ and the affiliations should be 1. School of Public Administration, Yanshan University, Qinhuangdao City, Hebei Province, China, 2. HEKRI of Marine Economy and Coastal Economic Zone, Hebei Normal University of Science and Technology, Qinhuangdao City, Hebei Province, China, 3. College of Transportation Engineering, Dalian Maritime University, Dalian City, Liaoning Province, China. Please download this article again to view the correct version. The originally published, uncorrected article and the republished, corrected articles are provided here for reference.

## Supporting information

S1 File Originally published, uncorrected article. (PDF)

S2 File Republished, corrected article. (PDF)
