# Supplementary material for: Correction: The spatial spillover effect and its attenuation boundary of urban economy on port efficiency
Source: PLoS One. 2026 Jul 31;21(7):e0355262. doi: 10.1371/journal.pone.0355262 (PMC13426986; doi:10.1371/journal.pone.0355262)
Supplement: S2 File — (PDF) [file pone.0355262.s002.pdf]

PLOS ONE

RESEARCH ARTICLE

The spatial spillover effect and its attenuation boundary of urban economy on port efficiency

Deng Zhao[1,2], **Xu Dongmei**[1], **Zhou Yutao**[3], **Daun Wei**[3*]

1 School of Public Administration, Yanshan University, Qinhuangdao City, Hebei Province, China, **2** HEKRI of Marine Economy and Coastal Economic Zone, Hebei Normal University of Science and Technology, Qinhuangdao City, Hebei Province, China, **3** College of Transportation Engineering, Dalian Maritime University, Dalian City, Liaoning Province, China

* duanwei106@aliyun.com

Check for updates

Abstract

Cities are commonly recognized as the immediate hinterland of ports and play a crucial role in fostering the sustainable development of ports. Therefore, it is imperative to investigate the influence of cities on ports. By employing panel data from 2001 to 2021 for both ports and cities in the Bohai Rim region, this study examines the spatial spillover effect of urban economy on port efficiency using the spatial error model (SEM). The findings show that urban economies have a significant spatial spillover effect on port efficiency, but this effect diminishes across different spatial matrices. In particular, the geographical matrix demonstrates a stronger spatial spillover effect of the urban economy on port efficiency. These research findings help to establish a collaborative mechanism for port-city development and provide useful insights for government management decision-making.

OPEN ACCESS

Citation: Zhao D, Dongmei X, Yutao Z, Wei D (2024) The spatial spillover effect and its attenuation boundary of urban economy on port efficiency. PLoS One 19(6): e0304973. https://doi.org/10.1371/journal.pone.0304973

Editor: Jiachao Peng, Wuhan Institute of Technology, CHINA

Received: November 15, 2023

Accepted: May 20, 2024

Published: June 5, 2024

Copyright: © 2024 Zhao et al. This is an open access article distributed under the terms of the Creative Commons Attribution License, which permits unrestricted use, distribution, and reproduction in any medium, provided the original author and source are credited.

Data availability statement: All relevant data are within the manuscript and its Supporting Information files.

Funding: This work was supported by the Hebei Key Research Institute of Humanities and Social Sciences at Universities[HYYB202304]; S&T Program of Qinhuangdao [202201B040]; Hebei Social Science Development Research Project in 2023[20230302028];Science Research Project of Hebei Education

1. Introduction

Port is an important infrastructure for urban development and a driving force in reshaping economic structure. It is not only a hub node for the intersection of land and sea as well as a gateway port for the city to open to the outside world, but it is also an important location for promoting the spatial agglomeration of flow factors [1,2]. The deepening of economic globalization has promoted the development of port functions from traditional water and land transportation node to the global supply chain center integrating logistics, commerce, industry and modern service industry [3], making ports an important engine for urban economic development and a force support for reshaping urban spatial structure [4,5].

A port city is a special type of city with the dual attributes of land and sea. Its special functions make it generally regarded as the port's direct hinterland, serving as a distribution hub for people flow, cargo flow, capital flow, technology flow and information flow [6]. Geographer Kautz pointed out that the port location is determined by the development of a city, and the economic scale and vitality of a city are the basis for the development of a port, as well as an important force for the expansion of a port and the improvement of its functions [7]. The

Department[BJS2024067];National Social Science Foundation Cultivation Project of Yanshan University [2023PY010], the host was Deng Zhao, who mainly took responsibility for the research design, formal analysis, manuscript preparation, etc.

Competing interests: There is no conflict of interest in the submission of this manuscript, and the manuscript is approved by all authors for publication, in whole or in part.

development of ports and cities has gradually transformed port cities into important hubs for global industrial transfer, playing a vital role in promoting port city development.

Ports and cities have a complementary relationship. On the one hand, ports' development promotes urban economic growth; on the other hand, cities provide talents, goods, funds, information, and technology for port development and play an important role in promoting port scale expansion, functional improvement, and efficiency improvement. With a weak global economy and disruptions caused by the COVID-19 epidemic, the role of the urban economy in supporting port development has diminished, forcing ports to enhance their port strength by improving operational efficiency and reducing operating costs [8–10]. However, we must also recognize the significant role of the urban economy in promoting port development and improving port efficiency [11–13]. Therefore, in the context of economic globalization, strengthening research on the impact of urban economies on port efficiency is crucial to understanding the new development model of port-city relations and the sustainable development of ports.

In recent years, driven by national strategies such as the coordinated development of the Beijing-Tianjin-Hebei region, the Shandong Peninsula Blue Economic Zone, the Northeast revitalization, and so on, ports and cities of the Bohai Rim have become more closely linked, and the scales and functions of ports and cities have continued to expand and improve. At the same time, the complex competitive relationships and significant regional differentiation within the Bohai Rim port group have gradually led to extensive research on the relationship between ports and cities in academic circles [14–16]. Driven by national strategies, the development of ports and cities in the Bohai Rim region will enter a new stage of development. Research on urban economy and port efficiency in the Bohai Rim region will further enrich the existing research perspectives and theories on port-city relations [13,17], which have significance for promoting the development of port cities in the region.

Therefore, this study, based on the theory of the port-city relationship, uses standard deviation ellipse and spatial econometric models to explore the spatial spillover effect and attenuation boundary of urban economy on port efficiency in order to provide a reference for the coordinated development of ports and cities in the Bohai Rim region. The innovation points and theoretical contributions of the research are mainly reflected in two aspects: (1) The research analyzes the coupling relationship between urban economy and port efficiency based on the standard deviation ellipse tool, verifies the spatial dependence between urban economy and port efficiency, and proposes novel concepts for port-city coupling research. (2) Based on the coupling of urban economy and port efficiency, the spatial econometric model is used to analyze the spatial spillover effect of urban economy on port efficiency, which enriches research on the impact of urban economy on ports and provides support for the coordinated development of port-city coupling.

The paper proceeds as follows. In Section 2 we review the literature relevant to the topic. Section 3 describes materials and methods. Section 4 includes analysis and discussion of empirical results. Section 5 is conclusions.

2. Literature

The relationship between port and city

As the special resource of the city, the port promotes the growth of the urban economy. The deepening and development of the urban economy constantly improve the functional system of the port. There is a complicated functional relationship between the port and the city in terms of space, function, economy, culture, and so on, which has gradually attracted the attention of academia. In the 1960s, relevant scholars began to propose a model of the interaction

between the port and the city from the perspective of location theory [18–20]. Thereafter, Hoyle [21,22] proposed the spatial interaction mechanism of port, industry and city from the perspective of port industrialization and regionalization. In the 1990s, with economic globalization and the development of modern shipping technology, the relationships between ports and cities became more complicated, and researchers began to explain the spatial and economic relationships between ports and cities from an urban perspective [23–27]. Research on port-city relations has shown that the interaction between ports and cities is the main manifestation of the evolution of port-city relationships, specifically manifested in the driving role of ports in cities. City, as a supporting platform, provides human, material, and financial support for port development [6,28–30]. For example, Bottasso et al. [31] believe that port-related industries have a strong pulling effect on cities and point out that ports not only affect local cities but also affect neighboring cities; Ferrari et al. [32] believe that scale expansion of urban industry and trade can release port-related functions to a certain extent. With the evolution of port-city relationships, scholars' research hotspots have gradually shifted from analyzing port-city relationships from the perspectives of coordinated development of port-city [33] and port-city interface [34] to solving problems of integrated development of port-city [35].

The relationship between port and urban economy

The dependence of the port and the economy is a direct reflection of the relationship between the port and the city. Studies have shown that port can not only reduce transportation costs, stimulate urban economy, and promote employment, but also produce agglomeration effect [31,36–38]. On the contrary, the urban economy can not only expand the scale of port [26], improve the port infrastructure, and accelerate the spatial agglomeration of production factors [32,39–41], but also provide financial, information, technology and other services for the port, affecting the future development direction of the port [42,43]. For example, the port infrastructure investment can promote urban economy increase, and urban economy promote the expansion of port throughput [27,41]. In addition, the location and transportation advantages of the port can promote the agglomeration of port industry and infrastructure construction, and promote the growth of urban economy [44,45]. Specifically, throughput and economy, as important indicators of port, are often used to measure the relationship between port and city [46,47]. Porto et al. [40] studied the relationship between port activities and economy, and discovered the importance of economy to port activities and regional infrastructure. Wang and Tan found that local economic construction and port construction promote each other [48]. Homosombat et al. [49] analyzed the impact of regional economic transformation on port and found that the relocation of factories had a negative impact on ports in the Pearl River Delta, and the biggest beneficiary was Hong Kong Port. Christofakis et al. used principal component analysis, cluster analysis and factor analysis to study the impact of the economic crisis on Greek port activities, and found that the economic crisis led to a decline in port traffic [50]. Peng et al. found that port trade has a significant impact on regional economic development, and the relationship between operational port trade and regional economic development has been effectively improved [51].

The relationship between port efficiency and urban economy

Port efficiency is an important indicator that reflects the optimal allocation of port resources and port competitiveness. In the context of port resource optimization and integration, improving port efficiency is very important for the government and operators, because it helps to enhance the comprehensive competitiveness of the port and realize the optimal allocation of port resources [52,53]. Therefore, research on port efficiency has gradually become

an issue of widespread concern among scholars. In recent years, there are many studies on port efficiency measurement based on DEA and SFA [9,54–58]. For example, Wanke et al. [59,60] used DEA method to measure the efficiency of major Brazilian ports and found that Brazil's port capacity is insufficient, but public-private partnership has a positive impact on port scale efficiency. Ju et al. found that competition among China's coastal ports is fierce, but competition improves the operational efficiency of ports [61]. Perez et al. found that specialized and large-scale ports are more efficient, and cooperation between ports of different specializations and ports of different scales is conducive to the coordinated development of ports [62]. In addition, scholars try to explore the influencing factors of port efficiency from the perspective of management and policy [63–66]. These studies suggest that port technical efficiency is positively related to the privatization of port operations [67,68]. For example, Tongzon and Wanke pointed out that the participation of the private sector in port operation management can improve port and infrastructure efficiency [9,69]. In contrast, other studies believe that human capital and production technology play significant roles in improving port efficiency [70,71]. Port efficiency is an important indicator for improving port productivity and is crucial to urban economic growth [72,73]. For example, Ayesu et al. found that port efficiency and port throughput promoted economic growth in Africa [74]. He and Xu analyzed the spatial network characteristics and influencing factors of port performance, and found that the port economy has a significant impact on the spatial correlation and spillover of port performance [75].

Generally, most previous literature focuses on the study of port-city relationships, port efficiency and influencing factors. However, in the era of economic globalization, port efficiency has become an important force in promoting urban economic growth, and urban economy plays an important role in upgrading port infrastructure and production technology [76]. However, there is a lack of research on the relationship between urban economy and port efficiency, especially the impact of urban economy on port efficiency [77].

In addition, traditional research on port efficiency is limited, as is the analysis of the relationship between port efficiency and economic growth. At the same time, there are few studies analyzing the spatial spillover effects of urban economy on port efficiency from an urban perspective. Therefore, this study uses standard deviation ellipse and spatial econometric models to analyze the coupling relationship between urban economy and port efficiency and the spatial spillover effect of urban economy on port efficiency from 2001 to 2021. This study can provide a reference for the coordinated development of port cities and the continuous improvement of port efficiency.

3. Materials and methods

Study area

The Bohai Rim region refers to the economic region composed of some coastal areas surrounding the Bohai Sea and the Yellow Sea, mainly including Beijing, Tianjin, Hebei Province, Shandong Province, Liaoning Province, and 43 prefecture-level cities (Fig 1). The Bohai Rim region has a land area of 521,600 square kilometers, a population of approximately 250 million, and a regional GDP of 21.67 trillion. It is the third engine of China's economic growth after the Yangtze River Delta and the Pearl River Delta [78]. The Bohai Rim port group is an important gateway for external exchanges in Northeast China, North China and Northwest China. It is an important port group for "the transmission of coal from northern to southern China" and "the transmission of grain from northern to southern China" and serves regional economic development (Fig 1). In 2022, the cargo throughput of Bohai Rim ports is 4.457 billion tons, and the container throughput is 75.52 million TEU, accounting for 43.99% and

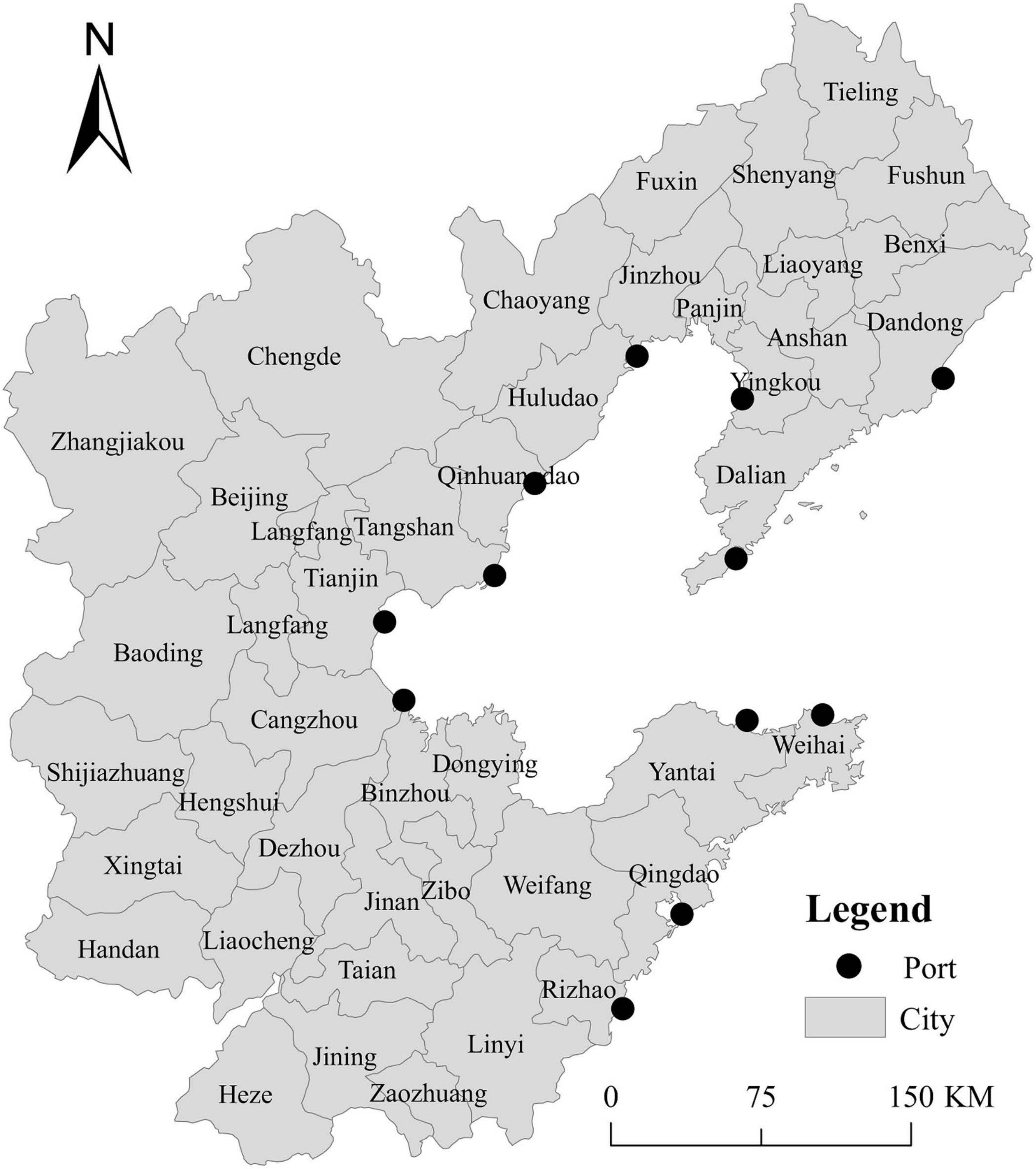

Fig 1. Distribution of ports and cities in the Bohai Rim region. (Note: The basic map came from a public map from the standard map service website (http://bzdt.ch.mnr.gov.cn/) of the Ministry of Natural Resources of China).

https://doi.org/10.1371/journal.pone.0304973.g001

28.96% of the national share respectively. The ports in the Bohai Rim region are relatively densely distributed, with diverse port scales and functional types. The regional differentiation within the port group is relatively obvious, and the competition and cooperation relationships between ports are relatively complex. Therefore, it is typical and representative to conduct research on the impact of urban economy on port efficiency in the Bohai Rim region.

Methods

Spatial econometric model. According to the First Law of Geography, everything has spatial correlation, and this correlation has obvious spatial distance attenuation characteristics. Therefore, the spatial econometrics is widely applied to the study of spatial interaction, and the spatial econometric model can be expressed by the spatial lag model and the spatial error model [79].

The spatial econometric model is a statistical model used to analyze spatial data. Since it takes into account spatial correlation and heterogeneity, it is usually used to reveal and analyze the spatial distribution characteristics and interrelationships of spatial data [79]. Spatial econometric models usually include spatial lag models, spatial error models and spatial Durbin models. LeSage and Pace believe that there are spatial correlation and heterogeneity factors in production activities, and regional development will be affected by different factors [80]. As special types of regional space, the development of ports will promote urban economic growth, and urban economic growth will provide support for port development.

Therefore, in order to explore the spatial characteristics and mutual relationships between ports and cities, spatial econometric models are selected to study the spatial spillover effect of urban economy on port efficiency. The specific production function formula is:

$$PE_{it} = \alpha + \alpha_1 lnGDP_{it} + \alpha_2 lnInvi_{it} + \alpha_3 lnFore_{it} + \alpha_4 lnRoad_{it} + \lambda WPE_{it} + \varepsilon,$$

$$\varepsilon = \lambda W\varepsilon + \mu \tag{1}$$

Where α is a constant term, and ε is an error term; i and t represent port and time, respectively; W represents a $n\times n$ order spatial weight matrix; λ represent spatial autocorrelation coefficient; *PE*, *GDP*, *Invi*, *Fore* and *Road* represent port efficiency, urban economy, investment level, trade level, collecting and distributing level, respectively; α_1,α_2,α_3,α_4 represent the elasticity coefficients of *PE*, *GDP*, *Invi*, *Fore* and *Road* respectively. To eliminate the influence of heteroscedasticity on the estimation results of the model, we conducted logarithmic processing for *PE*, *GDP*, *Invi*, *Fore* and *Road* variables respectively.

The spatial weight matrix is the basis of spatial econometric analysis. We use geographic distance matrix, economic and distance composite matrix to compare and analyze the differences in the impact of urban economy on port development under different spatial weight matrices. The geographic distance matrix and economic and distance composite matrix formula as follows,

$$W_{dis} = \begin{cases} 1/d_{ij}^2 & ,d_{ij} \geq d \\ 0 & ,d_{ij} < d \end{cases} \tag{2}$$

where d_{ij} is the geographic distance between port city i and port city j. We use the reciprocal square of the geographic distance between the two cities as the weight. The advantage of this method is that it fully considers the impact of geographic distance on the port, and the mutual influence

between port cities that are close in geographical distance but not adjacent is considered. The economic and distance composite matrix as follows,

$$W_c = W_{dis} \times diag(\frac{\overline{E_1}}{\overline{E}}, \frac{\overline{E_2}}{\overline{E}}, \cdots, \frac{\overline{E_n}}{\overline{E}}) \quad (3)$$

where W_c is the geographic distance matrix, $\overline{E_i}$ is the mean value of the economic variables of the port city i during the investigation period, and $\overline{E}$ is the mean value of all economic variables during the investigation period. This paper selects economic and distance composite matrix to measure the spatial effect of urban economy on port efficiency.

According to the First Law of Geography, spatial correlation gradually decreases with increasing distance. In order to test whether the spatial correlation of urban economy to port efficiency follows the geographical distance attenuation theory, this paper assumes that the geographical distance between ports and cities is $[d_{min}, d_{max}]$, and τ is the progressive distance from d_{min} to d_{max}. This model is specifically expressed as follows,

$$W_d = d_{\min}, d_{\min} + \tau, d_{\min} + 2\tau, \cdots, d_{\max} \quad (4)$$

where $W_d = [W_{ij}, d]_{n \times n}$ is the weight matrix,

$$W_{ij}, d = \begin{cases} e^{-d_{ij}/d_{\min}} & , d_{ij} \geq d \\ 0 & , d_{ij} < d \end{cases} \quad (5)$$

The spatial distance attenuation matrix is designed to test whether the gradual expansion of the distance between the spatial units participating in the spatial regression results in a gradual attenuation of the spatial correlation coefficient. As a result, by combining the geographic distance matrix and the composite matrix, the spatial spillover effects of the urban economy on port efficiency are estimated for distances of 50 km, 100 km, 200 km, 300 km, and 400 km, and the regression coefficient and Z value are recorded [81].

Spatial correlation test. Moran Index can determine whether the port efficiency is affected by spatial autocorrelation. The specific formula is as follows,

$$Moran's\ I = \frac{\sum_{i=1}^{n}\sum_{j=1}^{n} W_{ij}(X_i - \bar{X})(X_j - \bar{X})}{S^2 \sum_{i=1}^{n}\sum_{j=1}^{n} W_{ij}} \quad (6)$$

where $S^2 = \sum_{i=1}^{n}(X_i - \bar{X})^2$, $\bar{X} = \frac{1}{n}\sum_{i=1}^{n} X_i$, X_i, n and W_{ij} represent the observation of the i port (namely the port comprehensive strength), the total number of ports, and the spatial weight matrix, respectively.

Standard deviational ellipse. The standard deviation ellipse method is one of the classic methods for analyzing spatial distribution characteristics. Due to its advantages in revealing spatial distribution, it is often used in the fields of geography, economics and management. The standard deviation ellipse is based on the spatial distribution of elements and the distribution characteristics of spatial analysis elements. Therefore, spatial data analysis methods based on geographical information have become an important method for current spatial statistical analysis [82]. The standard deviation ellipse represents the distribution characteristics of geographical elements with parameters such as center store, major semi-axis, minor semi-axis, azimuth angle and spread range, and has certain advantages in quantifying the spatial distribution of urban economy and port efficiency. Therefore, this paper uses the standard deviation ellipse method to analyze the spatial characteristics of the coupling and

coordination of urban economy and port efficiency in the Bohai Rim region. The specific formula of the standard deviation ellipse is as follows:

$$\overline{X_w} = \frac{\sum_{i=1}^{n} w_i x_i}{\sum_{i=1}^{n} w_i}, \overline{Y_w} = \frac{\sum_{i=1}^{n} w_i y_i}{\sum_{i=1}^{n} w_i} \tag{7}$$

$$\tan\theta = \frac{(\sum_{i=1}^{n} w_i^2 \bar{x}_i^2 - \sum_{i=1}^{n} w_i^2 \bar{y}_i^2) + \sqrt{(\sum_{i=1}^{n} w_i^2 \bar{x}_i^2 - \sum_{i=1}^{n} w_i^2 \bar{y}_i^2)^2 + 4\sum_{i=1}^{n} w_i^2 \bar{x}_i^2 \bar{y}_i^2}}{2\sum_{i=1}^{n} w_i^2 \bar{x}_i^2 \bar{y}_i^2} \tag{8}$$

$$\sigma_x = \sqrt{\frac{\sum_{i=1}^{n} (w_i \bar{x}_i \cos\theta - w_i \bar{y}_i \sin\theta)^2}{\sum_{i=1}^{n} w_i^2}}$$
$$\sigma_y = \sqrt{\frac{\sum_{i=1}^{n} (w_i \bar{x}_i \sin\theta - w_i \bar{y}_i \cos\theta)^2}{\sum_{i=1}^{n} w_i^2}} \tag{9}$$

$$S = \pi \sigma_x \sigma_y \tag{10}$$

In Formula (7), $\overline{X_w}$ and $\overline{Y_w}$ are coordinates of center of gravity, and w_i represent weight. In Formula (8), θ is the azimuth angle of the ellipse, $\bar{x}_i$ and $\bar{y}_i$ represent the coordinate deviation from each research object location to the average center. In Formula (9), σ_x and σ_y represent the standard deviation along the x-axis and y-axis respectively. Formula (10) represents the area of an ellipse.

Variable selection and data collection

Variable selection. According to the assumptions of the spatial econometric model, the specific structure is as follows:

1. Port Efficiency (*PE*): Calculated using the DEA model. In view of the availability of data, this study, based on existing research [9,55,58,83], selects the number of port berths used for production, the number of berths above 10,000 tons, and the length of wharf used for production as input indicators, and port cargo throughput and container throughput as output indicators.
2. Urban economy (*GDP*): Urban economic development can provide more adequate public services and public productions for port development, laying the foundation for improving port efficiency. In this paper, the urban economy is measured by GDP. In order to eliminate the impact of prices on data, the GDP deflator is used to calculate the actual GDP with 2001 prices as the base period.
3. Investment level (*Invi*): As a capital-intensive industry, investment in port infrastructure construction is an important means to improve port efficiency. The investment level in this paper is measured by fixed asset investment.
4. Trade level (*Fore*): The improvement of trade level can not only promote the introduction of advanced knowledge and management experience but also promote competition among ports and promote ports to strengthen R&D and improve production efficiency. In this paper, the trade level is described by the ratio of the total volume of import and export trade to GDP.
5. Accessibility (*Road*): The land transportation infrastructure is an important part of the port's collecting and distributing system, and the accessibility of land transportation plays

Table 1. Description of the main indicators.

Variable	Symbol	Mean Value	Standard Deviation	Minimum	Maximum
Port Efficiency	*PE*	0.675	0.242	0.129	1.000
Urban Economy	*GDP*	16.752	1.083	14.439	19.032
Investment Level	*Invi*	16.075	1.381	12.739	18.893
Trade Level	*Fore*	0.391	0.275	0.044	1.325
Accessibility	*Road*	9.329	0.803	7.212	10.777

https://doi.org/10.1371/journal.pone.0304973.t001

an important role in the internal and external connections of the port. This paper uses road freight volume to characterize port accessibility.

Data collection. This study adopted panel data on 12 port cities around the Bohai Sea from 2001 to 2021, which was drawn mainly from the China City Statistical Yearbook (2002–2022), Yearbook Ports of China (2002–2022), and the statistical yearbook and bulletin of each province and city. The sample of ports was based on the "Layout Plan of National Coastal Ports" (2006); Dandong Port, Dalian Port, Yingkou Port, Jinzhou Port, Qinhuangdao Port, Tangshan Port, Huanghua Port, Tianjin Port, Qingdao Port, Yantai Port, Weihai Port, and Rizhao Port were selected. Variable definitions and descriptive statistics are shown in Table 1.

4. Results and discussion

Spatial-temporal pattern of urban economy and port efficiency

In this section, we explore the spatial differences in urban economy and port efficiency in the Bohai Rim port system: firstly, the standard deviation ellipse of economy and port efficiency is drawn by using the standard deviation (SDE) tool in ArcGIS 10.0; secondly, the overlapping area of urban economy and port efficiency is calculated; finally, the overlapping area is used to measure the degree of spatial coupling between urban economy and port efficiency, the larger the value, the stronger the spatial coupling [84]. As presented in Fig 2, the coupling degree of city economy and port efficiency presents a trend of decreasing first, then increasing, and then decreasing, but the average degree of coupling is relatively high, which can be roughly divided into three stages. In the first stage (2001–2006), the degree of coupling between urban economy and port efficiency dropped from 95.39% to 89.42%, but the overall degree of coupling is still high, indicating that port efficiency at this stage still depends on the promotion of urban economy. In the second stage (2006–2011), the degree of spatial coupling between urban economy and port efficiency soared from 89.42% to 94.70%, indicating that urban economy is the driving force of port efficiency. In the third stage (2011–2021), the degree of coupling between urban economy and port efficiency dropped from 94.7% to 80.74%, indicating that the degree of coupling between urban economy and port efficiency has gradually weakened. That is, urban economy and port efficiency are still closely related, but the dependence of port efficiency on the urban economy has declined. In addition, it can be found that the urban economy and port efficiency are always misaligned in space, which is manifested as complex phased characteristics in time, which verifies the spatial dependence relationship between economic growth and port efficiency to a certain extent.

The spatial spillover effect of urban economy on port efficiency

Spatial correlation test. Before calculating the spillover effect of urban economy on port efficiency, we checked for the spatial correlation, unit root test and cointegration test between urban economy and port efficiency to ensure the accuracy of the regression results.

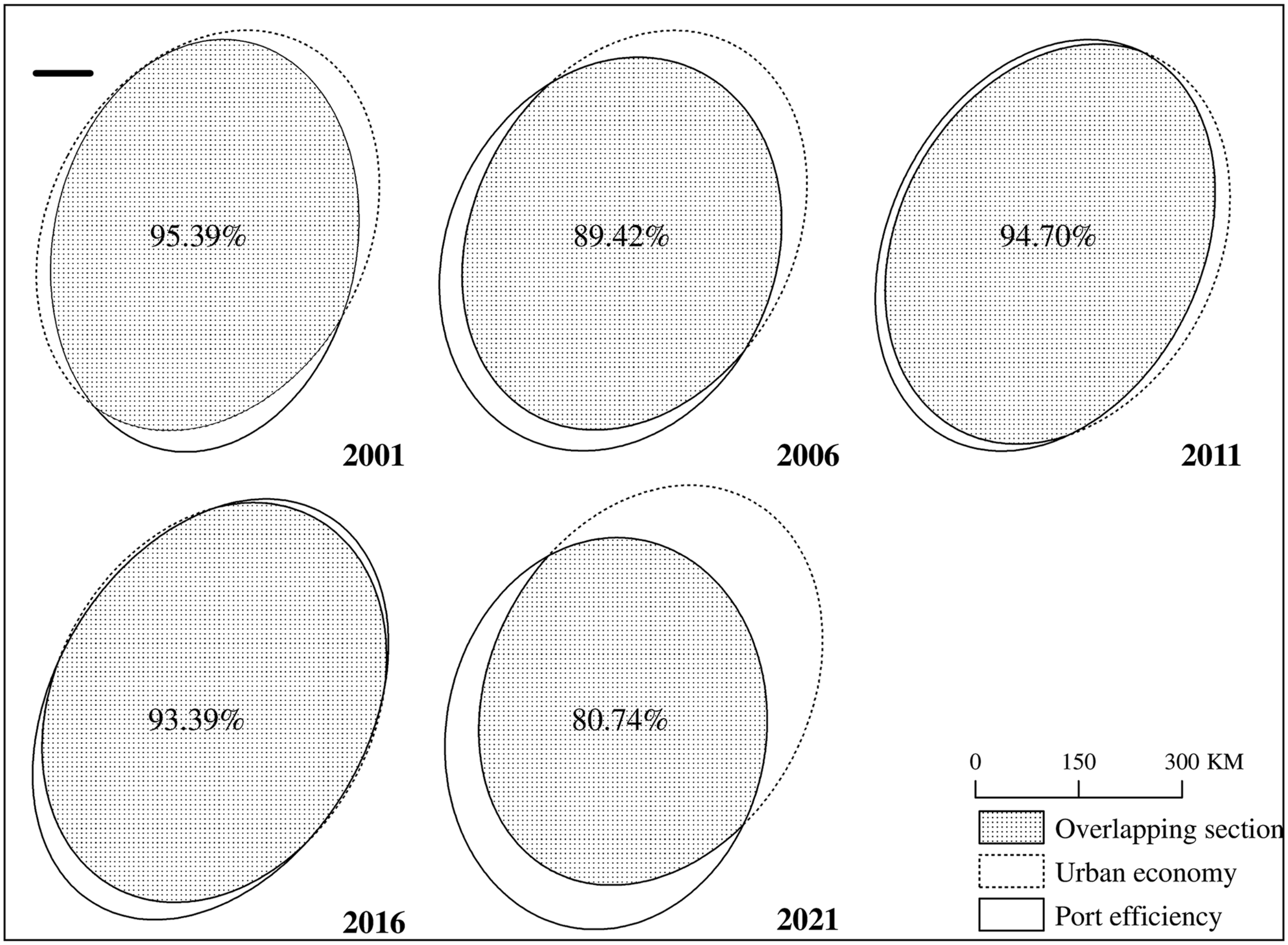

Fig 2. The Spatial-temporal pattern of urban economy and port efficiency from 2001 to 2021.

https://doi.org/10.1371/journal.pone.0304973.g002

Table 2 shows the spatial autocorrelation test results for the urban economy and port efficiency. Between 2001 and 2021, the Moran's I index of urban economy ranged from 0.3280 to 0.3367, while the Moran's I index of port efficiency ranged from 0.2602 to 0.3401. The average values for the two were 0.3346 and 0.3009, respectively, and both were significant at the 1% level, indicating that the urban economy and port efficiency in the Bohai Rim Region have a clear spatial correlation. The study also discovered that Moran's I has a small range of fluctuation between 2001 and 2021, indicating a relatively stable spatial correlation between urban economy and port efficiency.

Data test. We used Fisher-ADF, Fisher-PP, and LLC to test the unit root of the panel data and found that only ln *GDP* and ln *Invi* have panel unit roots and become stable in the first difference. Thus, a cointegration test is necessary. The Kao test and Pedroni test were used to test the cointegration relationship of the variables. The results showed a cointegration relationship between the variables at the 1% level, and regression analysis can be performed.

According to the selection method of Lesage and Pace spatial econometric model [80]. First, determine the spatial lag and spatial error terms through LM (Robust) test, and the LM (Robust) test results are displayed (Table 3): In the geographical distance matrix and the

Table 2. Spatial correlation tests of urban economy and port efficiency.

Urban Economy				Port Efficiency			
Year	Moran's I	Year	Moran's I	Year	Moran's I	Year	Moran's I
2001	0.3353***	2012	0.3280***	2001	0.3352***	2012	0.2971***
2002	0.3354***	2013	0.3325***	2002	0.2689***	2013	0.2944***
2003	0.3354***	2014	0.3333***	2003	0.2936***	2014	0.3005***
2004	0.3350***	2015	0.3340***	2004	0.2972***	2015	0.3010***
2005	0.3351***	2016	0.3365***	2005	0.2858***	2016	0.2911***
2006	0.3350***	2017	0.3367***	2006	0.3373***	2017	0.2602***
2007	0.3346***	2018	0.3366***	2007	0.3401***	2018	0.2768***
2008	0.3341***	2019	0.3361***	2008	0.3291***	2019	0.2742***
2009	0.3340***	2020	0.3362***	2009	0.3400***	2020	0.2802***
2010	0.3333***	2021	0.3366***	2010	0.3039***	2021	0.3099***
2011	0.3327***			2011	0.3032***		

Note: ***, **, and * reflect that Moran's I is significant at the level of 1%, 5% and 10% respectively.

https://doi.org/10.1371/journal.pone.0304973.t002

economic and distance composite matrix, the spatial error model is more suitable for testing the spillover effect of urban economy on port efficiency. Second, through Hausman tests fixed effects and random effects, and the test results reject the null hypothesis at the 1% level. Therefore, the spatial error model under fixed effects is selected to identify the spillover effects of urban economy and port efficiency.

Spatial spillover effect estimation. According to the regression results of the spatial econometric model in Table 4, the regression coefficient passes the significance level of 1%, implying that unobserved factors (residuals) that affect port efficiency will also adversely affect the efficiency of other surrounding ports. Under the stimulation of port development goals, many ports often engage in vicious competition through excessive construction. We found that the urban economy has a significant positive impact on the improvement of port efficiency in the geographical distance matrix and the composite matrix, and both have passed the significance test of 5%. More importantly, the driving effect of urban economy on port efficiency is significantly enhanced in the composite matrix. The regression coefficient value is expanded from 0.194 to 0.247, indicating that urban economy can indeed promote the improvement of port efficiency. The composite matrix is beneficial to eliminate the restraining effect of geographic distance, and further releasing the "driving potential energy" of the urban economy. In addition, the investment level, trade level, and city accessibility under the geographic distance matrix and the composite matrix have obvious spillover impacts on port efficiency.

Among them, the role of trade level in improving port efficiency in the geographical distance matrix is more obvious, indicating that the restriction of geographical distance does

Table 3. Spatial econometric model selection.

Test	Geographical distance matrix	Composite matrix
Hausman test	493.05***	163.73***
LM spatial error	17.991***	9.701***
Robust LM spatial error	49.954***	5.411**
LM spatial lag	2.635	7.326***
Robust LM spatial lag	34.599***	3.036*

Note: ***, ** and * indicate significant at the level of 1%, 5% and 10% respectively.

https://doi.org/10.1371/journal.pone.0304973.t003

Table 4. Estimation results of the spatial econometric model.

PE	Geographic distance matrix (SEM)		Composite matrix (SEM)	
	Coefficient	Z Value	Coefficient	Z Value
ln*GDP*	0.194***	5.01	0.247**	5.12
ln*Invi*	-3.537*	-1.89	-0.041***	-8.93
ln*Fore*	0.986***	18.45	0.023***	6.70
ln*Road*	0.181***	3.45	0.123***	4.93
λ	0.247***	3.40	0.082***	21.77
R^2	0.658		0.010	
Log-L	40.311		-528.016	
N	252		252	

Note: ***, **, * represent that the estimation results of the spatial econometric model are significant at the 1%, 5%, and 10% levels, respectively.

https://doi.org/10.1371/journal.pone.0304973.t004

not affect the promotion effect of trade level on port efficiency, while the embedding of the composite matrix limits the performance of trade level to a certain extent. Compared with the result of the geographic distance matrix, the regression coefficient of the trade level under the composite matrix decreased from 0.986 to 0.023, indicating that the joint effect of economy and geographical distance limits the promotion of port efficiency by trade level. The accessibility of urban transportation promotes the improvement of port efficiency, which means that the urban transportation accessibility increases by 1%, and the port efficiency increases by 0.181% and 0.123% respectively. At the same time, the investment level under the two matrices shows a significant negative effect for port efficiency, indicating that the improvement of port investment level cannot promote the improvement of port efficiency.

According to the spatial attenuation boundary estimation results of the impact of urban economy on port efficiency (Fig 3), it can be seen that the impact of urban economy on port efficiency in the geographical distance matrix presents a "V"-shaped spatial attenuation process of "first decreasing and then increasing". Specifically, when the geographical distance is between 50 and 200 km, the driving effect of urban economy on port efficiency decreases slowly. When the distance is over 100 km, the impact of the urban economy on port efficiency becomes smaller and smaller, and the further the distance, the stronger the inhibitory effect, indicating that the urban economy has a strong "polarization effect", and the further away from the economic center, the smaller the impact. When the geographical distance is between 200 and 400 km, the impact of urban economy on port efficiency is transformed into a "trickle-down effect", and the impact of urban economy on port efficiency gradually increases, indicating that economically developed cities can promote the economic development of neighboring areas through resource integration, financial support, information sharing, functional complementation, etc. Compared with the geographical distance matrix, the impact of urban economy on port efficiency in the composite matrix shows a "W"-shaped spatial attenuation process of "first decreasing, then increasing, then decreasing and then increasing". Specifically, when the geographical distance is between 50 and 150 km, the impact of urban economy on port efficiency shows a trend of first weakening and then strengthening. When the geographical distance is between 150 and 250 km, the inhibitory effect of the urban economy on port efficiency increases as the distance increases, indicating that the "polarization effect" of the urban economy becomes stronger and stronger under the combined effect of economy and geographical distance. This phenomenon accelerates the flow of production factors to economically developed cities, resulting in a spatial agglomeration effect, thereby

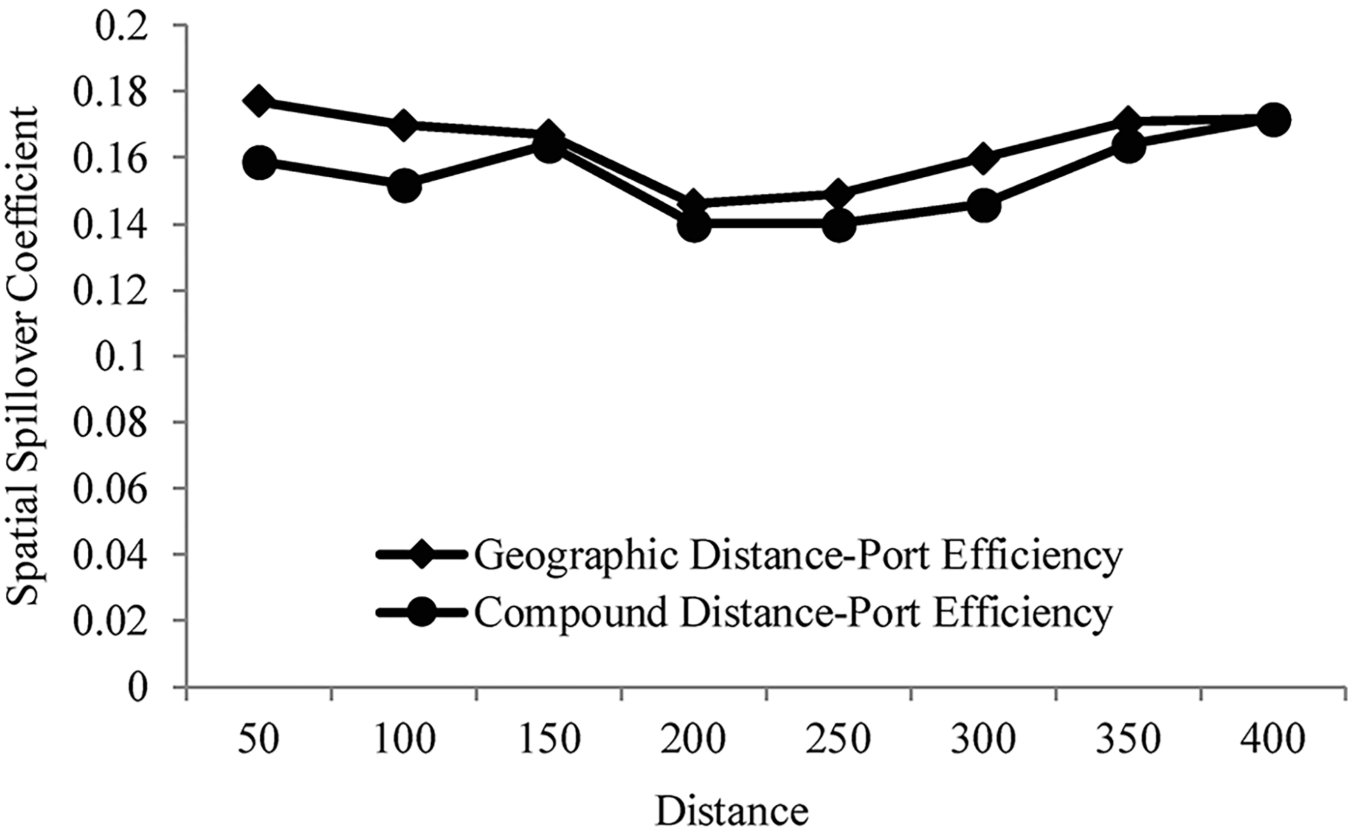

Fig 3. The spatial attenuation process of urban economy affects port efficiency.

https://doi.org/10.1371/journal.pone.0304973.g003

weakening the economic spillover effect on port efficiency in neighboring areas. When the geographical distance exceeds 250 km, the urban economy's role in promoting port efficiency continues to increase, and the impact is greatest at 400 km, indicating that economically developed cities promote port efficiency improvements with the support of technological innovation, infrastructure, capital investment, etc. When the geographical distance exceeds 400 km, the impact of urban economy on port efficiency is no longer significant in both matrices.

Discussion

Port efficiency is an important indicator for measuring port development and realizing the optimal allocation of port resources, and urban economy is an important platform to promote port development. The study discussed the spatial distribution pattern of urban economy and port efficiency in Bohai Rim region by using standard deviation ellipse tool and found that the degree of spatial coupling between urban economy and port efficiency is a significant difference, which must be linked to the urban economic development and the influence of economic policies.

One of the core findings of this study is to analyze the spatial coupling degree of urban economy and port efficiency based on the standard deviation ellipse method, which is different from previous research literature on the coupling relationship between ports and cities [6,34,85]. This study focuses on the overlapping section of the standard deviation ellipse to measure its degree of coupling, which provides support for theoretical and methodic innovation in port-city relationships. At the same time, we found that the degree of coupling between

urban economy and port efficiency showed a trend of first decreasing, then increasing and then decreasing, but the average degree of coupling was relatively high. The reasons for this phenomenon can be summarized as follows: At the beginning of the 21st century, the economic development of the Northeast China has faced numerous obstacles under the influence of the system and mechanism. The decline of hinterland economy has a certain impact on port development, leading to a decline in the degree of coupling between urban economy and port efficiency. From 2006 to 2011, with the support of national and local policies such as the revitalization of the Northeast China, the plan of the Shandong Peninsula Blue Economic Zone, and the development and opening of Tianjin Binhai New Area, the Bohai Rim region has gradually become a regional economic investment hotspot. In addition, the integration of port resources, and economic transformation and upgrading have promoted the improvement of the spatial coupling between urban economy and port efficiency. From 2012 to 2021, China's economy has gradually entered a new normal of slowing growth, structural adjustment and power transformation. At the same time, port development in the Bohai Rim region continues to slow down due to overcapacity, hinterland crossover, and vicious competition, which has led to a gradual decline in the degree of coupling between urban economy and port efficiency [86].

Another finding of this study is that urban economy has a significant spatial spillover effect on port efficiency, and this spatial spillover effect shows spatial attenuation characteristics in the geographical matrix and composite matrix dimensions. The study by Yuan et al. provided theoretical support to explain this phenomenon [81]. The perspective of this study is innovative. Compared with previous studies on port-city relationships and port efficiency [24,31,35,41,87,88], this study focuses on the spatial spillover effects of urban economy on port efficiency in the Bohai Rim region. On the one hand, as the high-quality development of urban economy continues to deepen, competition in coastal ports is also evolving towards high-quality development [89]. Against this background, it is both logical and in line with the goal of high-quality economic development in China to examine the spatial spillover effects of urban economy on port efficiency. On the other hand, this study studies the impact on port efficiency from an urban economic perspective, which is different from previous research on the impact of ports on urban economies [1,27] and offers new perspectives on port-city relationships.

In addition, there are differences in the spatial spillover effects of the urban economy on port efficiency in different matrices. The research results show that the urban economy in the geographical distance matrix has a stronger effect on improving port efficiency. At the same time, we found that the reason why urban economy can improve port efficiency is that economically developed areas can bring a sufficient supply of goods, complete infrastructure, and financial support to port development. The improvement of port efficiency depends on technological progress, and the acquisition of technology relies on strong financial support. Only economically developed areas can have the accumulation of capital and technology to promote the improvement of port efficiency [33].

Lastly, this study found that the impact of urban economy on port efficiency presents a spatial attenuation process. Research results on port efficiency have shown that market conditions, inland conditions and technical conditions may all have an important impact on changes in port efficiency [90]. However, few studies have explored the impact of economic development on changes in port efficiency, especially the spatial attenuation of the economic impact on port efficiency. Our research attempts to actively explore the impact of urban economy on port efficiency by changing the spatial distance in order to help port operators and government decision-makers formulate policies and urban spatial planning that are conducive to port development.

The research results have important policy implications for understanding the relationship between urban economy and port efficiency, promoting the integrated development of ports and cities, and improving port operation efficiency and development quality. Although our analysis mainly focuses on the Bohai Rim region, the relevant results provide certain empirical references for the development of ports in various countries around the world.

The limitations of this study will inspire future research. On the one hand, port efficiency needs to consider more micro-influencing factors, such as port machinery and equipment, port labor, port area, etc. However, data availability limits the refinement of our research. In the future, we will further measure port efficiency from a micro-perspective based on data acquisition. On the other hand, future research can explore the impact mechanism of urban economy on port efficiency from a provincial perspective to provide policy support for the high-quality development of China's ports.

5. Conclusions

The relationship between urban economy and port efficiency is the basis for promoting the coordinated development, transformation, and upgrading of ports and cities. Scientifically assessing the impact of urban economy on port efficiency has important practical significance. Therefore, this study selects panel data from 12 ports and cities in the Bohai Rim region from 2001 to 2021 and empirically analyzes the spatiotemporal pattern, spatial spillover effects, and attenuation boundaries of urban economy and port efficiency by constructing standard deviation ellipses and spatial econometric models. The main conclusions are as follows:

First, the relationship between urban economy and port efficiency presents an asymmetric pattern, which shows that the influence of urban economy on port efficiency has spatial dependence characteristics. Specifically, the spatial coupling degree of urban economy and port efficiency shows a trend of first decline, then rise and then decline, but the average degree of coupling is relatively high, and the degree of coupling between urban economy and port efficiency is manifested as complex phased characteristics in time. Second, urban economy has a significant spatial spillover effect on port efficiency, and this spatial spillover effect presents a "V"-shaped and "W"-shaped spatial attenuation process in the geographical distance matrix and composite matrix respectively, with an impact range from 50 km to 400 km.

Third, urban economic growth can promote the improvement of port efficiency, and the composite matrix can eliminate the inhibitory effect of geographical distance friction, releasing more of the "driving potential energy" of economic growth and promoting port efficiency.

This study has important policy significance for the implementation of port and urban planning and port efficiency optimization in the Bohai Rim region. (1) In theory, this study studies the relationship between urban economy and port efficiency, and the impact of urban economy on port efficiency. It enriches the existing research theory on port-city relationships and suggests novel directions for further investigation. (2) In practice, the empirical results of this study can help improve port efficiency and spatial planning of ports and cities, as well as provide guidance for port transformation and upgrading and port city integration construction.

Supporting information

S1 Data.
(XLSX)

Author contributions

Conceptualization: Deng Zhao, Xu Dongmei.

Data curation: Xu Dongmei, Daun Wei.

Formal analysis: Deng Zhao, Zhou Yutao.

Funding acquisition: Daun Wei.

Investigation: Deng Zhao, Xu Dongmei.

Methodology: Deng Zhao.

Project administration: Daun Wei.

Resources: Deng Zhao.

Software: Deng Zhao.

Supervision: Deng Zhao.

Validation: Deng Zhao.

Visualization: Xu Dongmei, Zhou Yutao.

Writing – original draft: Deng Zhao.

Writing – review & editing: Daun Wei.

References

1. Deng Z, Li ZF, Zhou YT, Chen X, Liang XX. Measurement and spatial spillover effects of port comprehensive strength: empirical evidence from China. Transport Policy. 2020; 99(2020):288–298.
2. Cao XS, Li SC. Spatio-temporal evolution of port opening in China's 40 years of reform and opening-up period. Plos One. 2019; 14(8):e0220912. https://doi.org/10.1371/journal.pone.0220912 PMID: 31404093
3. Robinson R. Ports as elements in value-driven chain systems: The new paradigm. Maritime Policy and Management. 2002; 29 (3):241–255.
4. Guo JK, Qin YF, Du XF, Han ZL. Dynamic measurements and mechanisms of coastal port-city relationships based on the DCI model: empirical evidence from China. Cities. 2020; 96 (1):102–440.
5. Wang XY, Wang N. The role of the port industry in China's national economy: An input-output analysis. Transport Policy. 2019; 78 (6): 1–7.
6. Guo JK, Qin YF. Coupling characteristics of coastal ports and urban network systems based on flow space theory: Empirical evidence from China. Habitat International. 2022; 126(8):102624.
7. Yang WY, Liang JS. Advanced economic geography. Beijing: Peking University Press; 1997.
8. Estache A, Gonzalez M, Trujillo L. Efficiency gains from port reform and the potential for yardstick competition: Lessons from Mexico. World Development. 2002; 30(4):545–590.
9. Cullinane K, Wang TF, Song DW, Ji P. The technical efficiency of container ports: comparing data envelopment analysis and stochastic frontier analysis. Transportation Research Part A: Policy and Practice. 2006; 40 (4):354–374.
10. Gonzalez M, Trujillo L. Efficiency measurement in the port industry: A survey of the empirical evidence. Journal of Transport Economics and Policy. 2009; 43(5):157–192.
11. Tongzon J, Heng W. Port privatization, efficiency and competitiveness: some empirical evidence from container ports. Transportation Research Part-A: Policy and Practice. 2005; 39(5): 405–424.
12. Chang YJ, Jo A, Choi KS, Lee S. Port efficiency and international trade in China. Transportmetrica A-Transport Science. 2020; 17(4):801–823.
13. Huang HY, Mo RB, Chen XC. New patterns in China's regional green development: an interval Malmquist-Luenberger productivity analysis. Structural Change and Economic Dynamics. 2021; 58(9):161–173.
14. Feng L, Notteboom T. Peripheral challenge by small and medium sized ports (SMPs) in multi-port gateway regions: the case study of northeast of China. Polish Maritime Research. 2013; 20(1):55–66.
15. Yang JL, Wang Grace WY, Li Kevin X. Port choice strategies for container carriers in China: a case study of the Bohai Bay Rim port cluster. International Journal of Shipping and Transport Logistics. 2016; 8(2):129–152.
16. Wang GWY, Zeng QC, Li K, Yang JL. Port connectivity in a logistic network: the case of Bohai Bay, China. Transportation Research Part E-Logistics and Transportation Review. 2016; 95(11):341–354.
17. Chen JH, Wang Z, Zhang FW, Park N, He XH, Yin WY. Operational efficiency evaluation of iron ore logistics at the ports of Bohai Bay in China: based on the PCA-DEA model. Mathematical Problems in Engineering. 2016; (2016):1–13. https://doi.org/10.1155/2016/9604819

18. Bird J. The Major Seaports of the United Kingdom. London; 1963. pp. 21–22.
19. Taaffe E, Morril R, Gould PR. Transport expansion in under developed countries. A comparative analysis Geographical Review. 1963; 53(4):503–529.
20. Hayuth Y. Containerization and the load center concept. Economic Geography. 1981; 57(2):161–176.
21. Hoyle BS, Pinder DA. City Port Industrialization and Regional Development. Belhaven, London; 1981.
22. Hoyle BS, Hilling D. Seaport System and Spatial Change: Technology, Industry and Development Strategies. Wiley, Chichesterm; 1984.
23. Notteboom T, Rodrigue J. Port regionalization: towards a new phase in port development. Maritime Policy & Management. 2005; 32(3):297–313.
24. Shan J, Yu MZ, Lee CY. An empirical investigation of the seaport's economic impact: Evidence from major ports in China. Transportation Research Part E-Logistics and Transportation Review. 2014; 69(9):41–53.
25. Heese M. Approaching the relational nature of the port-city interface in Europe: Ties and Tensions Between Seaports and the urban. Tijdschrift Voor Economische En Social Geografie. 2018; 109(2):210–223.
26. Feng GF, Wang QJ, Chang CP, Dong M, Wen J. Do economic growth and seaport throughput move together in port cities? International Journal of Transport Economics. 2018; 45(2):211–239.
27. Cong LZ, Zhang D, Wang ML, Xu HF, Li L. The role of ports in the economic development of port cities: panel evidence from China. Transport Policy. 2020; 90(2):13–21.
28. Ducruet C. A metageography of port-city relationship. Ports, cities, and global supply chains, Ashate; 2007. pp. 157–172.
29. Yeon JI, Hwang S, Jun B. Ports as catalysts: spillover effects of neighbouring ports on regional industrial diversification and economic resilience. Regional Studies. 2023; 1–18. https://doi.org/10.1080/00343404.2023.2268174
30. Ducruet C, Guerrero D. Inland cities, maritime gateways, and international. Journal of Transport Geography. 2022; 104(10):103433.
31. Bottasso A, Conti M, Ferrari C, Merk O, Tei A. The impact of port throughput on local employment: evidence from a panel of European regions. Transport Pololicy. 2013; 27(5):32–38.
32. Ferrari C, Percoco M, Tedeschi A. Ports and local development: evidence from Italy. International Journal of Transport Economics. 2010; 37(1):9–30.
33. Chen C, Lam JSL. Sustainability and interactivity between cities and ports: a two-stage data envelopment analysis (DEA) approach. Maritime Policy & Management. 2018; 45(7):944–961.
34. Van den Berghe K, Yacobs W, Boelens L. The relational geometry of the—interface: Case studies of Amsterdam, the Netherlands, and Ghent, Belgium. Journal of Transport Geography. 2018; 70(6):55–63.
35. Deng Z, Li ZF, Duan W. Research on the symbiosis of port and city based on symbiosis theory: empirical evidence from China's coastal port groups. International Journal of Shipping and Transport Logistics. 2023; 16(1–2):210–230.
36. Cohen JP. The broader effects of transportation infrastructure: spatial econometrics and productivity approaches. Transport Research Part E-Logistic and Transportation Review. 2010; 46(3):317–326.
37. Crescenzi R, Rodríguez-Pose A. Infrastructure and regional growth in the European Union. Papers Regional Science. 2012; 91(3):487–513.
38. Clark A, Hall PV. Maritime ports and the politics of reconnection. In: Desfor G, Laidley J., Stevens Q., and Schubert D (Eds.) Transforming Urban Waterfronys: Fixity and Flow. New York: Routledge; 2010. pp. 17–34.
39. Iannone F. The private and social cost efficiency of port hinterland container distribution through a regional logistics system. Transportation Research Part A: Policy and Practice. 2012; 46(9): 1424–1448.
40. Porto PCD, Ferrari C. Port and local economic development: what direction of the impacts? International Journal of Transport Economics. 2019; 46(4):27–44.
41. Song LL, Mi JN. Port infrastructure and regional economic growth in China: a Granger causality analysis. Maritime Policy & Management. 2016; 43(4):456–468.
42. Ducruet C, Itoh H. Regions and material flows: investigating the regional branching and industry relatedness of port traffics in a global perspective, Journal of Economic Geography. 2016; 16(4):805–830.
43. Zhao QY, Xu H, Wall RS, Stavropoulos S. Building a bridge between port and city: Improving the urban competitiveness of port cities. Journal of Transport Geography. 2017; 59(2):120–133.

44. Yang ZZ, Xiu QH, Chen DX. Historical change in the port and shipping industry in Hongkong and the underlying policies. Transport Policy. 2019; 82(10):138–147.
45. Wang JJ, Ma ZR. Port logistics cluster effect and coordinated development of port economy based on grey relational analysis model. Journal of Costal Research. 2019; 94:717–721.
46. Ding HC, Lian MR, Chen XY, Liu JM, Zhong ZC, Zhang YF, et al. Research on the correlation of port logistics and regional economic growth base on gray relational analysis method. Concurrency and Computation-Practice & Experience. 2019; 31(10):e4744.
47. Jaffe D. Neoliberal urbanism as 'Strategic Coupling' to global chains: Port infrastructure and the role of economic impact studies. Environment and Planning C-Politics and Space. 2019; 37(1):119–136.
48. Wang R, Tan QM. Dynamic model of port throughput's influence on regional economy. Journal of Coastal Research. 2019; 93:811–816.
49. Homosombat E, Ng Adolf K, Fu XW. Regional Transformation and Port Cluster Competition: The Case of the Pearl River Delta in South China. Growth and Change. 2016; 47(3):349–362.
50. Christofakis M, Tassopoulos A, Moukas B. Port activity evolution: the initial impact of economic crisis on major Greek ports. European Transport Research Review. 2013; 5(40):195–205.
51. Peng YJ, Tian Z, Wang C, Zhang HJ. An empirical study on the relationship between port trade and regional economic development based on VAR model. Ccamlr Science. 2018; 25(4):355–360.
52. Woo SH, Stephen P, Anthony KCB. Port Evolution and Performance in Changing Logistics Environments. Maritime Economics & Logistics. 2011; 13(3):250–277.
53. Soraya HG, Ramon NS, Pablo CM. Port allocative efficiency and port devolution: a study for the Spanish port authorities (1992–2016). Maritime Policy & Management. 2022; 49(1):39–61.
54. Tongzon J. Efficiency measurement of selected Australian and other international ports using data envelopment analysis. Transportation Research Part A-Policy and Practice. 2001; 35(2):107–122.
55. Cullinane K, Song DW, Wang TF. The application of mathematical programming approaches to estimating container port production efficiency. Journal of Production Analysis. 2005; 24(1):73–92.
56. Huang HY, Mbanyele W, Fan SS, Zhao X. Digital financial inclusion and energy-environment performance: What can learn from China. Structural Change and Economic Dynamics. 2022; 63(12):342–366.
57. Srisurin P, Pimpanit P, Jarumaneeroj P. Evaluating the long-term operational performance of a large-scale inland terminal: A discrete event simulation-based modeling approach. Plos One. 2022; 17(12):e0278649. https://doi.org/10.1371/journal.pone.0278649 PMID: 36469508
58. Wiegmans B, Witte P. Efficiency of inland waterway container terminals: stochastic frontier and data envelopment analysis to analyze the capacity design and throughput efficiency. Transportation Research Part A-Policy and Practice. 2017; 106(12):12–21.
59. Wanke PF, Barbastefano RG, Hijjar MF. Determinants of efficiency at major Brazilian port terminals. Transport Reviews. 2011; 31(5):653–677.
60. Wanke PF, Barros CP. Public-private partnerships and scale efficiency in Brazilian ports: evidence from two stage DEA analysis. Socio-Economic Planning Science. 2015; 51(9):13–22.
61. Ju SM, Xie J, Tang HL. The impact of competition on operational efficiency of ports: Empirical evidence from Chinese coastal port-listed companies. Research in Transportation Business and Management. 2023; 46(1):100939.
62. Perez I, Gonzalez MM, Trujillo L. Do specialisation and port size affect port efficiency? Evidence from cargo handling service in Spanish ports. Transportation Research Part A-Policy and Practice. 2020; 138(8):234–249.
63. Cullinane K, Song DW, Gray R. A stochastic frontier model of the efficiency of major container terminals in Asia: assessing the influence of administrative and ownership structures. Transportation Research Part A-Policy and Practice. 2002; 36(8):743–762.
64. Ramon NS, Pablo CM. The impact of public reforms on the productivity of Spanish ports: a parametric distance function approach. Transport Policy. 2012; 24(11):99–108.
65. Yuen ACL, Zhang AM, Cheung WM. Foreign participation and competition: a way to improve the container port efficiency in China? Transportation Research Part A-Policy and Practice. 2013; 49(3):220–231.
66. Ju SM, Liu N. Efficiency and its influencing factors in port enterprises: empirical evidence from Chinese port-listed companies. Maritime Policy & Management. 2015; 42(6):571–590.
67. Wang GWY, Knox KJ, Lee PTW. A study of relative efficiency between privatized and publicly operated US ports. Maritime Policy & Management. 2013; 40(4):351–366.

68. Serebrisky T, Sarriera JM, Suarez-Aleman A, Araya CBG, Schwartz J. Exploring the drivers of port effiency in Latin American and the Caribbean. Transport Policy. 2016; 45(1):31–45.
69. Wanke PF. Physical infrastructure and shipment consolidation efficiency drivers in Brazilian ports: a two stage network-DEA approach. Transport Policy. 2013; 29(9):145–153.
70. Thai VV, Yeo GT, Park JY. Comparative analysis of port competency requirements in Vietnam and Korea. Maritime Policy & Management. 2016; 43(5):614–629.
71. Song YW, Liu HW. Internet development, economic level, and port total factor productivity: an empirical study of Yangtze River ports. International Journal of Logistics-Research and Applications. 2020; 23(4):375–389.
72. Gurpinar N, Balcioglu HB. Impact of Famagusta Port Efficiency on North Cyprus Economic Development. Revista De Cercetare Si Interventie Social. 2018; 60(3):143–156.
73. Aguero-Tobar MA, Gonzalez-Araya MC, Gonzalez-Ramirez RG. Assessment of maritime operations efficiency and its economic impact based on data envelopment analysis: A case study of Chilean ports. Research in Transportation Business and Management. 2023; 46(1):100821.
74. Ayesu EK, Sakyi D, Darku AB. Seaport efficiency, port throughput, and economic growth in Africa. Maritime Economics & Logistics. 2023; 25(3):479–498.
75. He YY, Xu XF. Spatial correlation network structure of port performance and its drivers: A case study of Chinese coastal ports. Ocean & Coastal Management. 2023; 244(10):106780.
76. Choi SJ, Kim GS, Kim B. Economic efficiency of the Korean container terminals: a stochastic cost frontier approach. Journal of Korea Trade. 2022; 26(3):23–44.
77. Notteboom T, Coeck C, Van Den Broeck J. Measuring and explaining the relative efficiency of container terminals by means of Bayesian stochastic frontier models. International Journal of Maritime Economics. 2000; 2(2):83–106.
78. Yu Z, Di QB. Conceptualisation, estimation, and empirical analyses of land-sea convergenomics: A case study on Bohai Economic Rim cities. Plos One. 2022; 17(9):e0274707. https://doi.org/10.1371/journal.pone.0274707 PMID: 36126053
79. Anselin L. Spatial econometrics: a companion to theoretical econometrics. Basil Blackwell, Oxford; 2001.
80. LeSage J, Pace RK. Introduction to Spatial Econometrics. CRC Press, Florida; 2009.
81. Yuan HX, Liu YB. Financial agglomeration and green development efficiency: a two-dimensional perspective based on level and efficiency. Science Research Management. 2019; 40(12):126–143.
82. Wang XL, Wang L, Zhang XR, Fan F. The spatiotemporal evolution of COVID-19 in China and its impact on urban economic resilience. China Economic Review. 2022; 74(8):101806. https://doi.org/10.1016/j.chieco.2022.101806 PMID: 35601194
83. Huang HY, Mbanyele W, Wang FR, Zhang CX, Zhao X. Nuding corporate environmental responsibility through green finance? Quasi-natural experimental evidence from China. Journal of Business Research. 2023; 167(11):114147.
84. Yang ZC, Wu YX, Wang FF, Chen AC, Wang YX. Spatial-temporal differences and influencing factors of coupling coordination between urban quality and technology innovation in the Guangdong-Hong Kong-Macao Greater Bay Area. Plos One. 2023; 18(9):eo289988. https://doi.org/10.1371/journal.pone.0289988 PMID: 37733790
85. Zhou YT, Li ZF, Duan W, Deng Z. The impact of provincial port integration on port efficiency: Empirical evidence from China's Coastal Provinces. Journal of Transport Geography. 2023; 108(4):103574.
86. Deng Z, Duan W, Zhou YT. Analysis of port Transportation functions based on the structure of cargo types. Applied Spatial Analysis and Policy. 2023; 16(1):437–459.
87. Cullinane K, Song DW. A stochastic frontier model of the productive efficiency of Korean container terminals. Applied Economics. 2003; 35(3):251–267.
88. Pagano AM, Wang GWY, Sánchez OV, Ungo R. Impact of privatization on port efficiency and effectiveness: results from Panama and US ports. Maritime Policy & Management. 2013; 40 (2):100–115.
89. Liu PD, Pan Q, Zhu BY. Dynamic evolution of green total factor productivity growth of China's coastal ports. Maritime Policy & Management. 2023. https://doi.org/10.1080/03088839.2023.2205845
90. Cheon SH, Dowall DE, Song DW. Typology of long-term port efficiency improvement paths: Malmquist total factor productivity for World container ports. Journal of Infrastructure Systems. 2009; 15(4):340–350.
